# A comparison study of Zika virus outbreaks in French Polynesia, Colombia and the State of Bahia in Brazil

**DOI:** 10.1038/s41598-017-00253-1

**Published:** 2017-03-21

**Authors:** Daihai He, Daozhou Gao, Yijun Lou, Shi Zhao, Shigui Ruan

**Affiliations:** 1 Department of Applied Mathematics, The Hong Kong Polytechnic University, Hung Hom, Kowloon, Hong Kong (SAR) China; 2 Mathematics and Science College, Shanghai Normal University, Shanghai, 200234 China; 3 Department of Mathematics, University of Miami, Coral Gables, FL 33146 USA

## Abstract

Zika virus (ZIKV) disease outbreaks occurred in French Polynesia in 2013–2014 and in Brazil and Colombia in 2015–2016, respectively. Using our recently developed ZIKV disease model, we simulated the reported ZIKV infection cases from French Polynesia, Colombia and the State of Bahia of Brazil. Moreover, we estimated that the infection attack rates were 78.0% (95% confidence interval (CI): 63.5–86.3%) in French Polynesia which closely matches a previous serological study; 20.8% (95% CI: 1.1–50.0%) in Colombia which suggests that the attack rate was most likely less than 50%; and 32.4% (95% CI: 2.5–94.2%) in the State of Bahia in Brazil which suggests that the attack rate is unidentifiable with monthly data in Bahia. Furthermore, we found that the association of precipitation and ZIKV outbreak was more evident in Colombia than the other two places. These results are helpful for us to understand the possible evolution, to control the on-going outbreaks, to prevent the potential geographic spread, and to study the ecological and epidemiological characteristics of ZIKV.

## Introduction

An outbreak of Zika virus (ZIKV) hit French Polynesia in 2013–14 with more than 32,000 suspected cases^1–4^. In a serological survey, Cauchemez *et al.* ^4^ estimated that the infection attack rate of ZIKV among the 6–16 years old in French Polynesia was 66% (95% confidence interval (CI): 62–70%), compared to an overall infection attack rate 94% (95% CI: 91–97%) obtained in ref. 5 by fitting a compartmental model to the weekly cases (26 weeks) from six major archipelagos in French Polynesia. Even though the incidence rate among children seems significantly lower than adults (see Fig. 2 in the Zika Epidemiological Report ref. 6), the discrepancy between the two estimates seems too large to reconcile.

**Figure 1:**
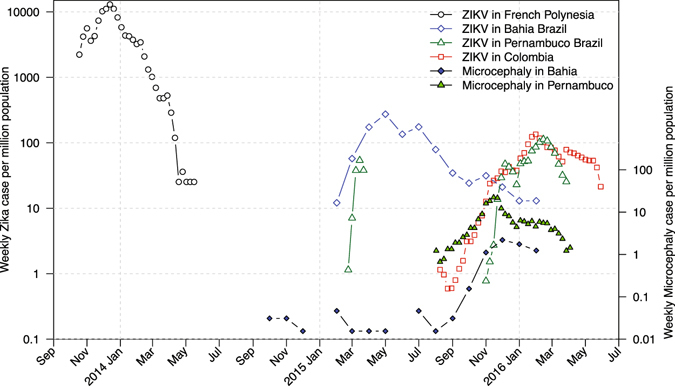
Scaled ZIKV cases and microcephaly cases. ZIKV data from French Polynesia (November 2014 to May 2015), the States of Bahia (February 2015 to February 2016) and Pernambuco (March-April 2015, November 2015 to April 2016) in Brazil, Colombia (August 2015 to June 2016), and microcephaly data from Bahia (July 2015 to February 2016) and Pernambuco (August 2015 - April 2016). All data are weekly except for the State of Bahia in Brazil, which were monthly and had been scaled by 1/4.25 to make them comparable. All time series are scaled by their respective population sizes. Microcephaly data for Colombia are not available.

In May 2015, a ZIKV outbreak in Brazil was first reported in the State of Bahia (Campos *et al.* ^7^). ZIKV subsequently spread to other states in Brazil as well as other countries and territories in the Americas, including Colombia^8, 9^. Data from the State of Pernambuco suggested that there were two waves of infection in Brazil. Apparently, the wave in early 2015 resulted in an observable number of microcephaly cases. Figure 1 presents the ZIKV and microcephaly cases from French Polynesia, states of Bahia and Pernambuco in Brazil, and Colombia. As of October 6, 2016, 196,976 and 95,412 suspected ZIKV infection cases had been reported in Brazil and Colombia, respectively^8^. Majumder *et al.* ^10^ presented a study to estimate the reproductive number of ZIKV epidemics in Colombia and obtained a basic reproductive number between 2.56 and 4.82. Towers *et al.* ^11^ used a compartmental model to fit the 2015 ZIKV epidemic data in Barranquilla, Colombia and estimated that ${ {\mathcal R} }_{0}=4.4$ (95% CI: 3.0–6.2) by Monte Carlo iteration. A recent review^12^ reported that the infection attack rate of ZIKV epidemic in the State of Bahia, Brazil up to the end of 2015 was larger than 2.5%.

There are various epidemiological studies on ZIKV outbreaks in other regions. Duffy *et al.* ^13^ conducted a serological study on the 2007 Yap Island ZIKV outbreak and reported that 73% (95% CI: 68–77%) of population (age ≥ 3 years) were infected during the epidemic. Funk *et al.* ^14^ built a compartmental model to investigate the 2007 ZIKV outbreak in Yap and inferred that the reporting rate was 3% (95% CI: 2–7%) and ${ {\mathcal R} }_{0}=4.3$ (95% CI: 3.1–6.1). Ellington *et al.* ^15^ estimated that the total infected ratio of ZIKV outbreak in Puerto Rico in 2016 was 25% with a range 10–70% by applying triangular distribution based on blood donor data for chikungunya.

All the above-mentioned studies were based on isolated ZIKV outbreaks. Since the ZIKV strains of some outbreaks were related^16, 17^, in this paper we will compare different outbreaks in order to understand the common as well as distinct epidemiological factors of ZIKV. These results will be helpful to study the evolution of ZIKV.

Seasonal drought periods have been associated with past West Nile virus (WNV) outbreaks^18^. Widespread drought in the spring followed by wetting during summer greatly increases the probability of a WNV epidemic in Florida^19^ and New Jersey^20^. To describe drought, Shaman *et al.* ^19^ used mean area water table depth (a measure of local land surface wetness) and Wang *et al.* ^21^ used mean annual precipitation. Johnson and Sukhdeo^20^ observed that early seasonal drought conditions (i.e., increased temperatures and decreased precipitation totals) are strongly associated with increases in yearly WNV infection rates in *Culex spp.* in New Jersey. Nevertheless, there are few studies relating the precipitation data with mosquito-borne disease data.

In our recent report^22^, a mathematical model was proposed to investigate the impact of mosquito-borne and sexual transmissions on the spread and control of ZIKV. Statistically, it was estimated that sexual transmission contributes 3.044% (95% CI: 0.123–45.73%) in the basic reproduction number and 4.437% (95% CI: 0.297–23.02%) in the attack rate. We also calibrated the model to the ZIKV epidemic data from Brazil, Colombia, and El Salvador, respectively. However, the data we used were only up to February 2016.

Now the one-year Zika virus infection datasets from both Brazil and Colombia^8^ are available, which are comparable to the dataset from the 2013–14 French Polynesia outbreak. We apply our recent modeling framework^22^ to simulate the weekly ZIKV cases (confirmed and suspected) from August 2013 to May 2014 in French Polynesia^2, 4^, from August 2015 to May 2016 in Colombia, and from February 2015 to February 2016 in the state of Bahia in Brazil^8^. The goal is to study the overall trend, common features, and distinct characteristics of ZIKV in these three outbreaks and to determine the effect of precipitation.

## Data

From Fig. 1, we can see that these ZIKV outbreaks reached their peaks in the beginning (or the first half) of a year. The population standardized incidence rates (cases per 1 million population) in Brazil and Colombia were smaller than that in French Polynesia. Data from the State of Pernambuco suggest that two ZIKV waves have occurred. The first wave seems highly under-reported, given the large amount of microcephaly cases reported there during the second wave and the substantial ZIKV wave in the State of Bahia in Brazil in early 2015, and the geographically adjacent relationship between Pernambuco and Bahia. The microcephaly rate is about 10 times higher in Pernambuco than in Bahia provided that the testing policies were similar in these two states. Thus, we would suspect that the early 2015 ZIKV incidence rate in Pernambuco should be 10 times high as in Bahia, if the risk of microcephaly due to ZIKV infection were the same in these two states. In late 2015, the testing effort was most likely strengthened in Pernambuco. In the following section, we use our model to fit the data from French Polynesia, the State of Bahia in Brazil and Colombia.

The French Polynesia wave and Colombia wave occurred roughly in the same time of a year, and both data are weekly. Thus we fit the two time series simultaneously in one framework to maximize the ratio of the data size to the number of model parameters. Since the Bahia data are monthly, we fit the data separately under the same assumption on mosquito abundance.

## Methods

Differed from^5^, we considered a time-dependent mosquito abundance which is more biologically realistic. Thus the instantaneous reproductive number is also time-dependent. Specifically, we assumed that the mosquito abundance contains two parts, a common trend and a distinct component associated with meteorological conditions. Given that the ZIKV lineages are the same in these outbreaks^16^, we assumed that the parameters and quantities are the same except for the population sizes, initial conditions, reporting ratio (due to different surveillance systems and health policies) and meteorological parameters. The common trend could be due to any other biotic or abiotic factors on mosquito population. We reduced the number of parameters by using a common trend. But we did not use the same trend in Bahia since the data are monthly, rather than weekly as in French Polynesia and Colombia.

We assumed that the mosquito abundance is time-varying by setting its ratio to the human population as *m*(*t*). Moreover, to represent the local environmental conditions for a specific region, this ratio is assumed to have two components

$$m(t)={m}_{{\rm{comm}}}(t)+{\xi }_{i}{p}_{i}(t),$$

where *m*_*comm*_(*t*) is the common flexible component (in the form of exponential of a cubic spline function) and *p*_*i*_(*t*) is the local precipitation with a parameter *ξ*_*i*_. We assumed that French Polynesia and Colombia share a common component with *n*_*m*_ nodes which are evenly distributed over the time duration. Following the steps in ref. 22, we first found the optimal flexibility in the common trend (number of nodes in the cubic spline, *n*_*m*_). Then we obtained the maximum log-likelihood estimates for the reproduction number, reporting ratio, and infection attack rate with the fixed *n*_*m*_. The reporting ratio is defined as the proportion of symptomatic cases that were reported, and the infection attack rate is defined as the proportion of population that were infected during the outbreak.

We downloaded monthly mean climatic data for the most populous city in each place (Tahiti in French Polynesia, Bogota in Colombia, Salvador for Bahia) from www.bbc.com/weather/. Since the seasonal fluctuations in temperature were much milder than in precipitation, we only focused on precipitation in this work. We used the *loess* function (Local Polynomial Regression Fitting) in R to convert monthly precipitation data to daily data and then incorporated the daily precipitation into our model simulations. Our model was simulated with a fixed step-size of 1 day using the Euler-multinomial integration method^23^.

## Results

**Figure 2:**
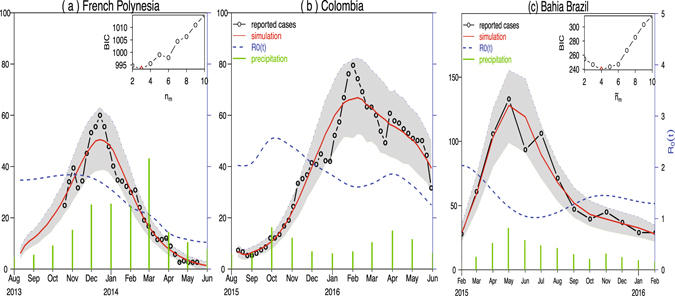
Fitting model to ZIKV cases in (**a**) French Polynesia in 2013–14; (**b**) Colombia in 2015–16; and (**c**) the State of Bahia in Brazil in 2015–2016. Black circle curves represent observed cases, red curves indicate the medians of 1000 simulations with estimated parameters, the shaded regions are the 95% ranges, and blue dashed curves show the estimated reproduction numbers. The insert shows the profile Bayesian Information Criterion (BIC) as a function of the number of nodes in the mosquito abundance.

**Figure 3:**
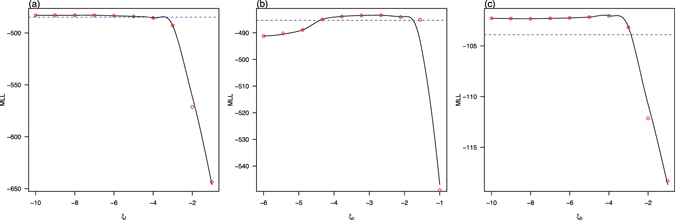
Maximum log-likelihood (MLL) as a function of parameter *ξ*_*i*_ for (**a**) French Polynesia, *ξ*_f_; (**b**) Colombia, *ξ*_c_; and (**c**) the State of Bahia in Brazil, *ξ*_b_. The red circles denote the estimated MLL at the given value of the control parameter. The black curves denote Local Polynomial Regression Fittings with a span of 0.5. The blue dotted lines indicate the thresholds of −$\frac{1}{2}{\chi }_{\mathrm{0.95,1}}^{2}$ from the maximum of the MLL. The maximum value of the black curve gives the maximum log-likelihood estimate of the control parameter, while the intersections of the two curves yield the 95% CI.

**Figure 4:**
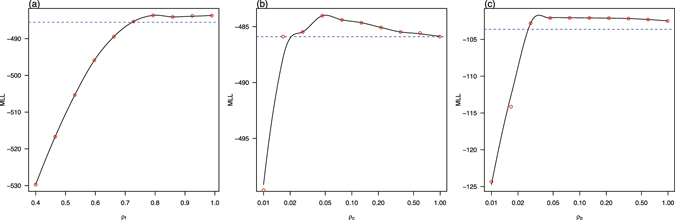
Maximum log-likelihood (MLL) as a function of reporting ratio *ρ* for (**a**) French Polynesia, *ρ*_f_; (**b**) Colombia, *ρ*_c_; and (**c**) the State of Bahia in Brazil, *ρ*_b_, respectively. The red circles denote the estimated MLL at the given value of the control parameter. The black curves denote Local Polynomial Regression Fittings with a span of 0.5. The blue dotted lines indicate the thresholds of −$\frac{1}{2}{\chi }_{\mathrm{0.95,1}}^{2}$ from the maximum of the MLL. The maximum value of the black curve gives the maximum log-likelihood estimate of the control parameter, while the intersections of the two curves yield the 95% CI.

We used our mathematical model (Gao *et al.* ^22^) to simulate the reported ZIKV cases from French Polynesia in 2013–14 (Fig. 2(a)), Colombia in 2015–16 (Fig. 2(b)) and the State of Bahia in Brazil in 2015–2016 (Fig. 2(c)). We found that the model simulations for French Polynesia and Colombia attain the smallest BIC at *n*_*m*_ = 3 (see inset panel of Fig. 2(b)). While for the State of Bahia in Brazil, since the data are monthly, we used a separate *m*_*comm*_(*t*), denoted as ${\tilde{m}}_{{\rm{comm}}}(t)$, and *ξ*_*b*_ *p*_*b*_(*t*), and found that the State of Bahia in Brazil attains the smallest BIC at ${\tilde{n}}_{m}=4$ (see inset panel of Fig. 2(c)). We showed the maximum log-likelihood as a function of the precipitation parameter *ξ* and reporting ratio *ρ* in the three regions in Figs 3 and 4. The estimated *ξ* has wide confidence intervals (containing zero) in French Polynesia and Bahia which suggests that the effect of precipitation is indistinguishable in these two places. This is different from Colombia, where the confidence interval of *ξ* does not contain zero. The estimated reporting ratio is higher with smaller confidence interval in French Polynesia than in the other two places.

We estimated an infection attack rate of 78.0% (95% CI: 63.5–86.3%) for French Polynesia which is largely in line with a previous estimate of 66% (95% CI: 62–70%) among 6–16 years old children obtained by Cauchemez *et al.* ^4^. According to^6^, the ZIKV incidence rates are significantly lower among children (younger than 15 years old) than in adults, which could explain our slightly higher estimates. We also applied our framework to the weekly archipelago level data in French Polynesia, with the weekly proportion of stations reporting, and obtained reasonable attack rates as well, 71.3%(95% CI: 67.4–94.1%) in Tahiti, 70.1% (95%CI: 66.3–92.5%) in Ile Sous, and 62.5% (95%CI: 59.2–82.5%) in other four archipelagos.

**Figure 5:**
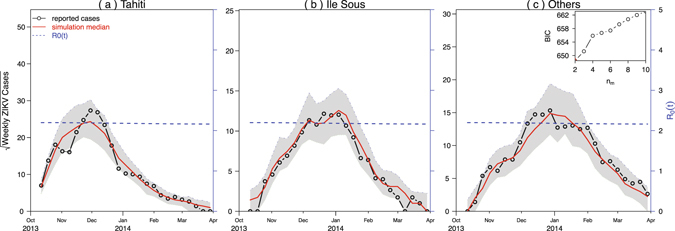
Fitting model to French Polynesia regional level ZIKV cases with the weekly proportion of stations reporting. In each region, the reporting ratio of symptomatic cases is the baseline reporting ratio (*ρ*) multiplied by the proportion of stations reporting in each week. We used *loess* to convert the weekly proportion of stations data into daily data.

Not only our estimated attack rates are more reasonable, but also the goodness-of-fit of our model works better than previous studies with the same number of parameters, see Fig. 5. This supports our estimates of other parameters. The estimated overall attack rate in Colombia from August 2015 to May 2016 was 20.8% (95% CI: 1.1–50.3%) which is substantially lower than that in the 2013–14 French Polynesia outbreak. Colombia has a population size of 48 million and a birth rate of 0.0189 per capita. Since the reported number of pregnant women infected with ZIKV as of the 33rd week of 2016 in Colombia was 18,363 ^6^, if the population is completely homogeneous and 18% of the ZIKV-infected pregnant women were detected^13^, then the attack rate was approximately

$$\mathrm{1.8363/(0.0189}\times \mathrm{4800)/0.18}\times \mathrm{100 \% }=\mathrm{11.25 \% }$$

which also indicates that the attack rate was low in Colombia. All other estimates (e.g., reproductive number) and assumptions match previous studies^5^.

For comparison, we list the estimates of reporting ratios and infection attack rates with 95% confidence intervals of these regions in Table 1. The reporting ratio could be as high as our estimate and the data quality is guaranteed. The difference between our estimate and previous serological study (age 6–16 year) in French Polynesia could be due to lower incidence rate among children than the population mean incidence rate^6^.

**Table 1:** Parameter estimates for French Polynesia, Colombia, and the State of Bahia in Brazil.

Region	Population	Reporting ratio *ρ*_*i*_	Infection attack rate	Precip. ln ξ_*i*_
French Polynesia	276,831	80.5% (72.8–100.0%)	78.0% (63.5–86.3%)	−7.27 (−10,−3.36)
Colombia	48,000,000	5.1% (2.1–100.0%)	20.8% (1.1–50.3%)	−2.82 (−4.38, −1.76)
Bahia Brazil	15,000,000	3.5% (2.7–100.0%)	32.4% (2.5–94.2%)	−4.27 (−10, −2.91)
Tahiti	178,100	95.4% (70.9, 100.0%)	71.3% (67.4%, 94.1%)	N/A
Ile Sous	33,100		70.1% (66.3%, 92.5%)	
Others	47,400		62.5% (59.2%, 82.5%)	

The 95% confidence intervals are given in the parentheses.

## Discussion

It is believed that the Brazil and Colombia ZIKV strain originated from French Polynesia^16, 17^. All three outbreaks (French Polynesia, Colombia, the State of Bahia in Brazil) took off in a relatively dry season when the monthly precipitation was low. The seasonal fluctuations of the air temperature were much milder than the precipitation, thus we focused on precipitation only. Our flexible model framework allowed us to test the impact of precipitation on the transmission of ZIKV. We found that the effects of the precipitation on mosquito abundance (thus ZIKV transmission) are not consistent across the three places. The strongest impact occurred in Colombia.

Since the effect of precipitation was not evident in French Polynesia, precipitation was not included in fitting regional level data. However, we took into account the weekly proportion of stations that reported cases as did in ref. 5. We achieved evidently better simulations (closer to observed cases with small confidence range) than in ref. 5. Moreover, our estimated attack rates are closer to previous serological study^4^.

Besides weekly (or monthly) ZIKV cases, other types of data (e.g., serological study) are needed to give more accurate estimate of the attack rate. At this stage, we can only conclude that the attack rates in Colombia and the State of Bahia in Brazil were most likely less than 50%.

The estimates of the attack rates and reporting ratios are very crucial in studying the evolution of ZIKV and in assessing the severity of an outbreak. The low attack rate in Colombia implies that parts of population were not infected during the 2015–16 ZIKV outbreak, hence a second wave of the epidemic could sweep the country. The lower attack rate in Colombia could partly be due to higher altitude and cooler weather than the other places.

To the best of our knowledge, this was the first attempt to fit these three outbreaks with a time-dependent mosquito abundance and to compare the ZIKV attack rates in these three regions. In the future, we believe that comprehensive studies on the biology/seasonality/distribution of mosquitoes in these places are needed, both directly on mosquitoes and indirectly through studies of other mosquito-borne diseases (such as dengue in these regions). The inhomogeneities of incidence rates across gender and age also deserve further studies.
